# Autophagy in adult stem cell homeostasis, aging, and disease therapy

**DOI:** 10.1186/s13619-025-00224-2

**Published:** 2025-04-10

**Authors:** Ke Zhao, Indigo T. C. Chan, Erin H. Y. Tse, Zhiyao Xie, Tom H. Cheung, Yi Arial Zeng

**Affiliations:** 1 Key Laboratory of Systems Health Science of Zhejiang Province, School of Life Science, Hangzhou Institute for Advanced Study, University of Chinese Academy of Sciences, Hangzhou, 310024 China; 2 New Cornerstone Science Laboratory, State Key Laboratory of Cell Biology, CAS Center for Excellence in Molecular Cell Science, Institute of Biochemistry and Cell Biology, Chinese Academy of Sciences, University of Chinese Academy of Sciences, Shanghai, 200031 China; 3 Division of Life Science, Center for Stem Cell Research, State Key Laboratory of Molecular Neuroscience, Daniel and Mayce Yu Molecular Neuroscience Center, HKUST-Nan Fung Life Sciences Joint Laboratory, the Hong Kong University of Science and Technology, Hong Kong, China; 4 Hong Kong Center for Neurodegenerative Diseases, Hong Kong, China

**Keywords:** Autophagy, Adult stem cell, Homeostasis, Aging, Cancer

## Abstract

Autophagy is a crucial cellular process that facilitates the degradation of damaged organelles and protein aggregates, and the recycling of cellular components for the energy production and macromolecule synthesis. It plays an indispensable role in maintaining cellular homeostasis. Over recent decades, research has increasingly focused on the role of autophagy in regulating adult stem cells (SCs). Studies suggest that autophagy modulates various cellular processes and states of adult SCs, including quiescence, proliferation, self-renewal, and differentiation. The primary role of autophagy in these contexts is to sustain homeostasis, withstand stressors, and supply energy. Notably, the dysfunction of adult SCs during aging is correlated with a decline in autophagic activity, suggesting that autophagy is also involved in SC- and aging-associated disorders. Given the diverse cellular processes mediated by autophagy and the intricate mechanisms governing adult SCs, further research is essential to elucidate both universal and cell type-specific regulatory pathways of autophagy. This review discusses the role of autophagy in regulating adult SCs during quiescence, proliferation, self-renewal, and differentiation. Additionally, it summarizes the relationship between SC aging and autophagy, providing therapeutical insights into treating and ameliorating aging-associated diseases and cancers, and ultimately promoting longevity.

## Background

Stem cells (SCs) are undifferentiated cells characterized by their capacity to self-renew and differentiate into various somatic cell types (Saba et al. 2021). Adult SCs, also known as somatic stem cells, play a vital role in tissue homeostasis and stem cell pool maintenance (Southard 2016; Ratajczak 2017). They are essential in repairing degraded and damaged tissues. Adult SC functions are highly regulated by the niche environment through paracrine, and endocrine signals (Ouspenskaia et al. 2016; Li et al. 2019; Fuchs and Blau 2020). The activation, proliferation, differentiation, and self-renewal processes are under tight control.

Adult SCs exhibit diversity in their differentiation potential and cell states, ranging from unipotent (e.g., epidermal SCs) to bipotent (e.g., hepatic progenitors) and multipotent (e.g., mesenchymal SCs), as demonstrated in lineage-tracing studies (Wabik and Jones 2015; Pu et al. 2023). Based on the turnover rate of specific tissues, Adult SCs may either remain continuously active or persist in a dormant state, which is a reversible G_0 _phase referred to as quiescence (Lee et al. 2016; Gala et al. 2022). Generally, in high-turnover tissues, adult SCs remain continuously active. For instance, hematopoietic stem cells (HSCs) (Yokomizo and Suda 2024) are responsible for replenishing naturally degrading blood cells, intestinal stem cells (ISCs) (Goto et al. 2022) repair the wear and tear of the intestinal lining, and skin stem cells (Gonzales and Fuchs 2017) in the epidermis consistently replace lost or damaged skin cells. However, recent work has identified rare quiescent SC subpopulations in high-turnover tissues, including + 4 intestinal SCs and hair follicle bulge SCs that contribute to regeneration under stress (Garcin et al. 2016; Gonzales and Fuchs 2017; Goto et al. 2022; Higa et al. 2022). Conversely, adult SCs in low-turnover tissues exist in a quiescent state. This includes muscle stem cells (MuSCs) found in skeletal muscle and neural stem cells (NSCs) in the nervous system (Verma et al. 2018; de Morree and Rando 2023). Quiescence is essential to enable the SC to endure metabolic stress and maintain its genomic integrity throughout its lifespan. Quiescent adult SCs only re-enter the cell cycle and activate in response to intrinsic and extrinsic stimuli, such as autophagy and injury, to contribute to extensive SC differentiation and tissue regeneration (Wang et al. 2016b; Urbán et al. 2019).

During aging, the functions of adult SCs become dysregulated. Their capacity to self-renew and differentiate diminishes, resulting in the exhaustion of the adult stem cell pool and impairment in tissue regeneration, thereby increasing the susceptibility of the affected tissue to age-related diseases (Keyes and Fuchs 2018; Cai et al. 2022). On the contrary, aberrant activation of adult SCs can cause uncontrolled proliferation and cancer development (Yao et al. 2016). Therefore, a thorough understanding of the comprehensive mechanisms that regulate adult SC functions will be imperative to promote longevity and prevent aging-associated disease progression.

Autophagy is a highly conserved cellular mechanism that selectively degrades damaged organelles (e.g., via mitophagy) and protein aggregates (e.g., via aggrephagy), while recycling cellular components to maintain proteostasis and metabolic flexibility (Levine and Kroemer 2019). While basal autophagic activity occurs constitutively, factors such as nutrient deprivation, energy deficiency, and oxidative stress contribute to increased autophagy (Lahiri et al. 2019). Recent studies have revealed that autophagy is involved in various aspects of SC regulation, encompassing shared and distinct pathways. Dysregulation of autophagy alters stem cell functions, contributing to aging and disease development (Boya et al. 2018; Trentesaux et al. 2020; Orhon et al. 2022). Pharmacological or genetic modulation of autophagy activity levels has been shown to effectively restore the proliferative/differentiation potential of aged SCs, demonstrating promising clinical applications in regenerative medicine. However, the complexity arises from the diverse roles of autophagy in regulating adult SC fate determination, which necessitates more systematic and in-depth investigations to fully elucidate the intricate relationship between autophagy and adult SC fate. This review summarizes the current research on the relationships between autophagy and the regulation of adult SCs in terms of quiescence, activation, cell fate determination, and differentiation. The review will also discuss the role of autophagy during adult SC aging, and its potential therapeutic applications to combat aging-associated diseases and cancers.

## Autophagy: functions and mechanisms

Autophagy is a highly conserved cellular process that involves the degradation of cytoplasmic components within lysosomes, playing an essential role in maintaining cellular homeostasis (Adelipour et al. 2022). The primary functions of autophagy include facilitating proteostasis and regulating energy homeostasis (Boya et al. 2018).

Autophagy mediates proteostasis by degrading misfolded and denatured protein clumps, referred to as protein aggregates, to achieve a balanced and functional proteome. When exposed to stresses such as heat shock and oxidative stress, proteins are misfolded and damaged, forming potentially toxic protein aggregates (Levine and Kroemer 2019). The recognition of these substrates involves specific autophagy receptors such as Sequestosome 1 (p62/SQSTM1) (Gatica et al. 2018).

Besides, autophagy serves as a key adaptive response to starvation (Kim and Lee 2014). During nutrient deprivation, autophagy is upregulated to catabolize non-essential proteins, lipids, and glycogen, thereby providing substrates to synthesize essential macromolecules for energy production and cellular survival (Kim and Lee 2014). For instance, time-restricted feeding (TRF) is a dietary intervention that restricts food intake and has been shown to induce autophagy and promote metabolic health (Xie et al. 2022). By activating the autophagy-related genes and enhancing the formation of autophagosomes, TRF maintains cellular homeostasis, reduces oxidative stress, mitigates age-related dysfunctions, and improves overall physiological functions, thereby playing a key role in promoting longevity (Hansen et al. 2018).

**Fig. 1:**
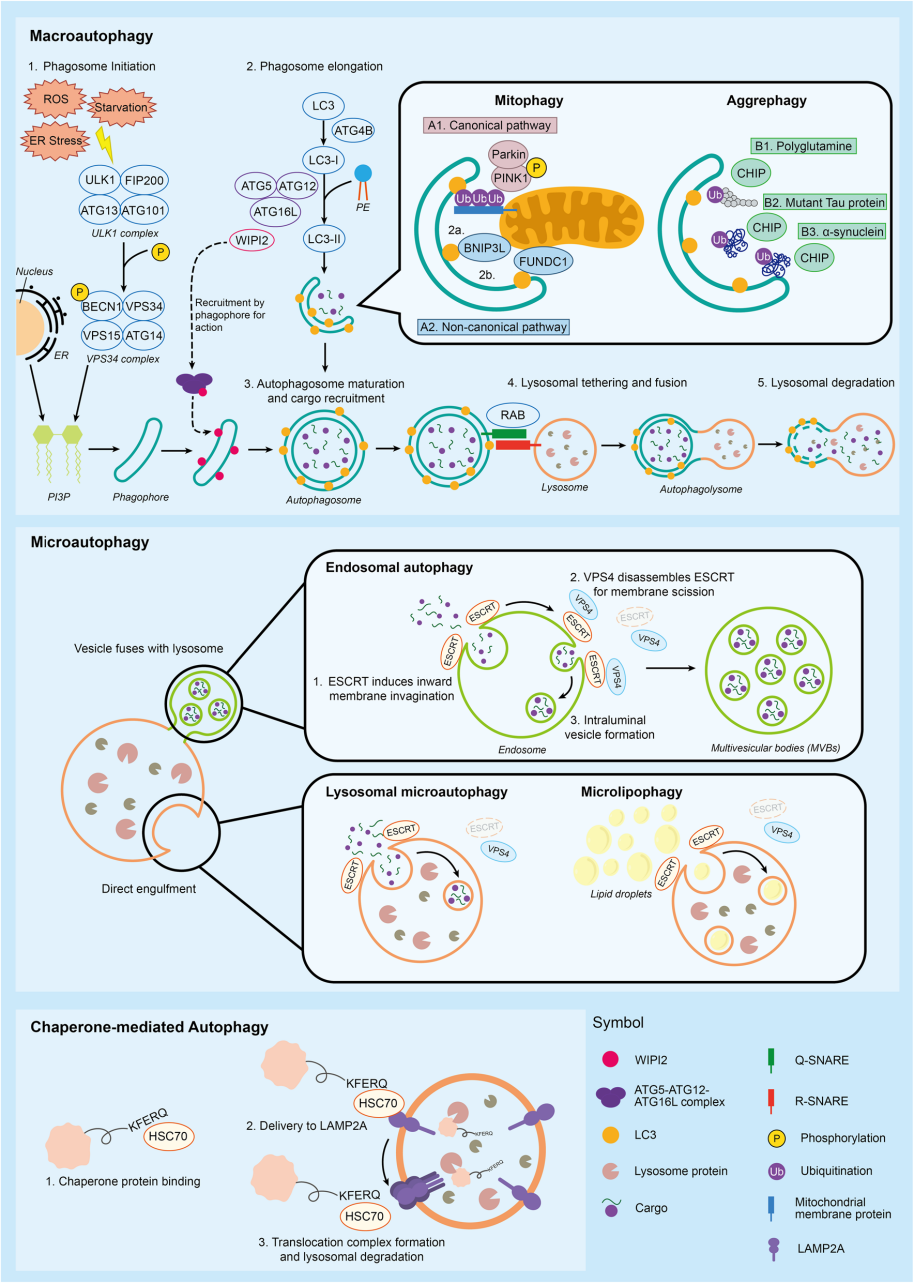
The process of macroautophagy, microautophagy, and chaperone-mediated autophagy. In macroautophagy, cargos are recruited by a double-membrane autophagosome, which then fuses with the lysosome to facilitate degradation. In microautophagy, cytoplasmic contents are directly engulfed by lysosomes or encapsulated by vacuolar membranes that protrude from the ER, endosome, and cell membrane, thereby fusing with the lysosome to facilitate degradation. Chaperone-mediated autophagy involves the recognition of proteins containing KFERQ motif by HSC70, leading to their translocation to lysosome for degradation

Autophagy is characterized into three main forms, namely macroautophagy, microautophagy, and chaperone-mediated autophagy (Fig. 1). The mechanisms and functions of these processes are distinctive, in which the pathways towards isolation and delivery of cellular cargoes to lysosomal degradation vary (Klionsky et al. 2021).

### Macroautophagy

Macroautophagy (hereafter referred to as autophagy) is the most widely studied form of autophagy. It involves the formation of a double-membrane structure known as the autophagosome, which envelops substrates and delivers them to lysosomes for degradation (Klionsky et al. 2021). The process of autophagy can be subdivided into phagophore initiation, vesicle elongation, autophagosome maturation, lysosomal fusion, and autolysosomal degradation (Klionsky et al. 2021; Rahman et al. 2022).

By intracellular or extracellular stimuli, including nutrient deprivation and oxidative stress, Unc-51 Like Autophagy Activating Kinase (ULK1) complex is activated and translocated to autophagosome formation sites, such as the periphery of the endoplasmic reticulum (ER) and cellular membrane, where the lipid supply is abundant. The ULK1 complex then phosphorylates the Vacuolar protein sorting 34 (VPS34) complex to enhance phosphatidylinositol 3-phosphate (PI3P) synthesis and thus initiates phagophore formation (Levine and Kroemer 2008; Polson et al. 2010; Ktistakis et al. 2011; Dooley et al. 2014). The ubiquitin-like conjugation system elongates phagophores and promotes the synthesis of double-membrane autophagosomes (Glick et al. 2010). WIPI2 (WD repeat domain, phosphoinositide interacting 2) links PI3P to the ubiquitin-like conjugation system by recruiting ATG proteins, such as the ATG5-ATG12-ATG16L complex, to the PI3P-constituted phagophore membranes (Rao et al. 2024). Microtubule-associated protein 1 light chain 3 (LC3) is first cleaved by ATG4B (Autophagy-related protein) to convert into LC3-I. The ATG5-ATG12-ATG16L complex then conjugates LC3-I to phosphatidylethanolamine (PE) to form LC3-II (Kroemer et al. 2010; Kaur and Debnath 2015). LC3-II binds to both the exterior and interior membranes of the autophagosome and plays a critical role in phagophore elongation, cargo recruitment, and, eventually, autophagosome formation (Li et al. 2020). Finally, RAB (Ras-associated binding) proteins, such as Rab7 and Rab2, recruit lysosomes into close proximity to the autophagosomes. Q-SNAREs (Soluble N-ethylmaleimide-sensitive factor attachment protein receptors) (e.g., STX17 and SNAP47) on the outer membrane of autophagosomes tether with R-SNAREs (e.g., VAMP7 and VAMP8) on the lysosomes, facilitating membrane fusion (Shen and Mizushima 2014; Wang et al. 2016c; Jian et al. 2025; Jian et al. 2024). Eventually, the cargoes encapsulated by the autophagosomes are degraded and recycled within the autolysosomes by hydrolytic enzymes, including SNXs complexes and Rab32/38 (Dikic and Elazar 2018; Mizushima 2018; Wu et al. 2024).

Macroautophagy can be further divided into selective autophagy targeting specific cargoes, such as mitophagy and aggrephagy. Mitophagy degrades damaged or dysfunctional mitochondria to maintain mitochondrial quality control. The clearance of damaged mitochondria prevents the accumulation of ROS (reactive oxygen species), a source of oxidative damage, and maintains the efficiency of oxidative phosphorylation to support ATP production (Chen et al. 2020). Mitochondrial depolarization, in which the ATP production is disrupted, induces mitophagy (Ding and Yin 2012). In the canonical mitophagy pathway, the PTEN-induced putative kinase 1 (Pink1)—parkin RBR E3 ubiquitin protein ligase (Prkn) pathway ubiquitinates the outer mitochondrial membrane proteins and recruits the mitochondria to LC3. Mitophagy can also be activated by the non-canonical pathways. For instance, BCL2/adenovirus E1B interacting protein 3-like (Nix/BNIP3L), BCL2/adenovirus E1B interacting protein 3 (BNIP3), and FUN14 domain containing 1 (FUNDC1) all contain the LC3-interacting region (LIR) which selectively target damaged mitochondria for degradation without ubiquitination (Fiesel et al. 2015; Shiba-Fukushima et al. 2017; Chu 2019). Dysregulation of mitophagy is associated with a variety of diseases, including neurodegenerative and cardiovascular diseases (Dikic and Elazar 2018).

Aggrephagy selectively degrades protein aggregates to clear misfolded and damaged proteins from the cells, protecting the cells from cytotoxicity. Aggrephagy involves the use of specific receptors or the recognition of ubiquitinated cargoes. For instance, polyglutamine, mutant Tau protein, and α-synuclein are ubiquitinated by E3 ubiquitin ligases such as CHIP (C-terminus of Hsc70 interacting protein) to facilitate degradation (Gatica et al. 2018; Øverbye et al. 2007). These aggregates are associated with neurodegenerative diseases such as Huntington's disease, which results from an abnormal expansion of CAG repeats that encode proteins with expanded polyglutamine tracts. Also, tauopathies are characterized by the accumulation of abnormal tau filaments, as seen in Alzheimer's disease and frontotemporal lobar degeneration. Whereas synucleinopathies like Parkinson's disease involve the aggregation of α-synuclein. Aggrephagy can be monitored in cellular and animal models using immunofluorescence, immunogold labeling, and filter trap assays to quantify aggregate levels (Petroi et al. 2012; Shahpasandzadeh et al. 2014; Kleinknecht et al. 2016).

### Microautophagy

Microautophagy refers to the direct engulfment of small portions of cytoplasmic components by lysosomes or vacuolar membranes derived from the ER, endosome, or cell membrane. Microautophagy does not involve the formation of large autophagosomes and is considered less common in higher eukaryotes; instead, it is primarily observed in yeast and plant cells (Mijaljica et al. 2011). In mammalian cells, ESCRT (Endosomal sorting complexes required for transport) plays a crucial role in microautophagy by mediating membrane invagination, vesicle formation, and scission, eventually transporting the engulfed cytoplasmic components to the lysosomes for degradation. For instance, CHMP4B (Charged Multivesicular Body Protein 4B) is a core subunit of the ESCRT complex which drives inward membrane invagination (Loi et al. 2019). Besides, VPS4 (Vacuolar Protein Sorting 4 Homolog A) promotes the disassembly of the ESCRT complex, thereby facilitating the scission and the formation of intraluminal vesicles (Sun et al. 2017). Endosomal autophagy, also known as microvesicular microautophagy, is a specific form of microautophagy that occurs at late endosomes. The process involves the ESCRT-mediated inward budding of endosomal membranes and VPS4-mediated membrane scission, forming intraluminal vesicles within late endosomes and subsequently transforming into multivesicular bodies (MVBs) (Mukaiyama et. al 2004). Eventually, MVBs are fused with lysosomes to facilitate lysosomal degradation. Lysosomal microautophagy, on the other hand, involves the ESCRT-mediated lysosomal membrane invagination and direct engulfment of cytosolic components. Microlipophagy is one of the specific forms of lysosomal microautophagy in which lipid droplets are directly uptaken and degraded by lysosomes (Vevea et. al 2015; Oku et. al 2017).

### Chaperone-mediated autophagy

Chaperone-mediated autophagy (CMA) is a highly selective and conserved autophagy process specifically targeting soluble cytosolic proteins (Dice 1990; Lescat et. al 2018). Proteins containing the KFERQ motif are recognized by heat shock cognate protein 70 (HSPA8/HSC70) and transported to Lysosomal receptor lysosomal associated membrane protein 2A (LAMP2A) on lysosomes (Kaushik and Cuervo 2018). The binding of the substrate proteins stimulates monomeric LAMP2A to assemble into a multimeric translocation complex, allowing the substrate proteins to enter the lysosome. Notably, this motif is also used for selective targeting of the ER in microautophagy (Sahu et al. 2011; Caballero et al. 2018). Therefore, additional criteria are needed to distinguish CMA from ER microautophagy, such as the use of specific inhibitors (Morozova et al. 2016).

### Current practice of autophagy assessment

In the study of autophagy, autophagic flux is a common measurement which encompasses the entire process of autophagy from the formation of autophagosomes to their fusion with lysosomes and subsequently to the degradation of their contents. Since autophagy is a dynamic process, measuring autophagic flux rather than just the number of autophagosomes can provide a more accurate assessment of autophagic activity (du Toit et al. 2018). The visualization of autophagic activity is primarily based on LC3. LC3 is converted from its cytosolic form (LC3-I) to its lipidated form (LC3-II), which is then integrated into the autophagosome membrane (Kabeya et al. 2000). While LC3-II is widely used to monitor autophagosomes, its interpretation requires complementary assays (e.g., TEM, flux analysis) due to non-specific autophagic localization (Klionsky et al. 2021).

There are several pharmacological agents which have been shown to alter autophagic activity (Table 1). Rapamycin induces autophagy by inhibiting mTORC1 (mammalian target of rapamycin 1 complex 1). Conversely, hydroxychloroquine/chloroquine (HCQ/CQ) and Bafilomycin A1 (BafA1) suppress autophagy by blocking the fusion of autophagosomes with lysosomes (Maycotte et al. 2012; Chude and Amaravadi 2017). The introduction of these drugs can aid in analyzing the changes in autophagy and autophagic flux. Furthermore, behavioral interventions remodel autophagy. For instance, starvation or dietary restriction, which limits nutrient uptake, is a physiological trigger of autophagy (Kuma et al. 2004; Mizushima et al. 2004; Morselli et al. 2010). Exercise is also known to stimulate autophagy in skeletal muscle (Egan and Zierath 2013). These strategies are valuable to investigating the responses and metabolisms of associated cells and tissues.

**Table 1:** Autophagy agonists/antagonists and their targets

**Autophagy Agonists**	**Targets**	**Refs**
Metformin	Activates AMPK, which directly activates the ULK1 complex and inhibits mTORC1, leading to autophagy induction	Meng 2015; Tanaka et al. 2019
Rapamycin	Binds to FKBP12, which in turn binds to mTORC1 and inhibits its activity, leading to autophagy induction	Frias et al. 2023
ABT-737	Induces autophagy by activating Beclin 1 as a BH3-mimetic compound	Pedro 2015
Salicylate	Activates autophagy by directly binding to and activating AMPK	Liu et al. 2023
Resveratrol	A Sirt1 activator that can induce autophagy through Sirt1 activation	Zhang et al. 2022
Urolithin A	A metabolite of ellagitannins that extends healthspan and lifespan and enhances mitophagy	Ryu 2016
CCCP (Carbonyl Cyanide 3-ChloroPhenylhydrazone)	Induces mitophagy by promoting the recruitment of Parkin to damaged mitochondria	Moskal 2020
**Autophagy Antagonists**	**Targets**	**Refs**
3-MA (3-Methyladenine)	Inhibits autophagy by inhibiting PI3K to block formation of autophagosomes	Liu et al. 2021
Bafilomycin A1	Inhibits lysosomal function and autophagosome-lysosome fusion	Mauvezin and Neufeld 2015
Chloroquine	Inhibits the fusion of autophagosomes with lysosomes	Mauthe 2018
Hydroxychloroquine	Similar to chloroquine, it inhibits lysosomal function and autophagosome-lysosome fusion	Kim 2021
T1742	Blocks the ATG5-ATG16L1 and ATG5-TECAIR interactions, inhibiting autophagy	Xiang 2023
Mdivi-1	A mitochondrial division inhibitor that can block mitophagy by inhibiting Drp1	Su et al. 2023

Autophagy has been implicated in a range of diseases, including neurodegenerative disorders, cancer, and metabolic diseases (Hara et al. 2006). Growing evidence indicates that autophagy not only contributes to the quiescence, proliferation, self-renewal, and differentiation of adult SCs, but also modulates SC aging and potentially the development of aging-associated diseases. Understanding how autophagy regulates the fate determination of adult SCs can offer insights into its role in disease pathogenesis as well as potential therapeutic opportunities.

## Adult stem cell regulation

Adult SCs are present in various tissues and possess the remarkable capacity to self-renew and differentiate into specialized cell types specific to their tissue origin. Adult SCs are pivotal in maintaining tissue homeostasis and facilitating tissue repair and replenishment (Gabut et al. 2020). The states of the adult SCs can be categorized into quiescence, activation, self-renewal, and differentiation, all of which are tightly regulated to ensure proper maintenance and transitions between states.

Stem cell quiescence is a reversible G_0 _state where adult SCs remain inactive and exhibit low metabolic activity (Cheung and Rando 2013). Quiescence is vital for enduring metabolic stress and safeguarding genomic integrity, allowing adult SCs to stay functional throughout the lifespan of the organism. Quiescent stem cells are characterized by low RNA content (Saba et al. 2021) and a compact heterochromatin structure to maintain low transcriptional activity (Dong et al. 2022). P53, Notch, Wnt and BMP signaling are the major pathways modulating stem cell quiescence, while the Forkhead box O (FOXO) family also plays a role in withstanding metabolic stress (de Morree and Rando 2023; Cheung and Rando 2013; Cheng et al. 2024).

In response to intrinsic and extrinsic signals, such as growth factors and environmental cues, quiescent adult SCs re-enter the cell cycle, activate, and proliferate. The transition involves a shift in chromatin conformation from heterochromatin to a more accessible euchromatin structure (Dong et al. 2022). While overall gene expression and protein synthesis levels increase, specifically, cell cycle progression-associated and proliferation-associated genes are upregulated. Besides, several types of adult SCs undergo metabolic reprogramming, transitioning from glycolysis to mitochondrial oxidative phosphorylation to support the heightened energy demands required for rapid cell division and synthesis of intracellular components (Cliff and Dalton 2017).

Following the expansion of the SC population, SCs either commit to differentiate into a specific lineage or return to the quiescent state. SCs can undergo symmetric division, where both daughter cells either differentiate or retain stem cell properties, or asymmetric division, where one daughter cell remains a stem cell while the other commits to differentiation (Evano et al. 2020). Self-renewal is crucial for maintaining the resident SC pool and preserving the potency for future lineage differentiation. Conversely, SC differentiation is essential to repopulate and repair the damaged or degraded tissues. For instance, transcription factors and niche-derived signals play critical roles in modulating HSCs towards particular differentiated cell lineages.

As organisms age, both the quantity and functionality of SCs diminish. The chromatin landscape of the quiescent SC is dysregulated. For instance, aged quiescent muscle SCs shift towards a chronically activated chromatin signature, altering gene expression patterns (Dong et al. 2022). SCs experience accumulated oxidative stress, compromised mitochondrial functions, and exhibit hallmarks of cellular senescence (Zeng et al. 2023). Concurrently, the SC niche is distorted, with the extracellular matrix becoming stiffer and its composition changes. These alterations collectively contribute to SC dysregulation, resulting in impaired tissue regeneration capacity. Collectively, aged SCs contribute to age-related diseases and cancer development.

The fundamental role of stem cells in maintaining tissue homeostasis and the profound impact of stem cell aging on overall physiology underscore the critical importance of elucidating stem cell mechanisms. Such knowledge is essential for developing targeted interventions to ameliorate aging phenotypes and improve tissue functions in aged individuals for a better healthspan. Recently, autophagy has been revealed to be a crucial cellular process that regulates multiple aspects of SC functions. Exploring the correlation between SC metabolism and autophagy provides an alternative angle in understanding tissue homeostasis and disease progression.

## Autophagy in quiescent stem cells

### Quiescent SCs exhibit various levels of autophagic activity

**Fig. 2:**
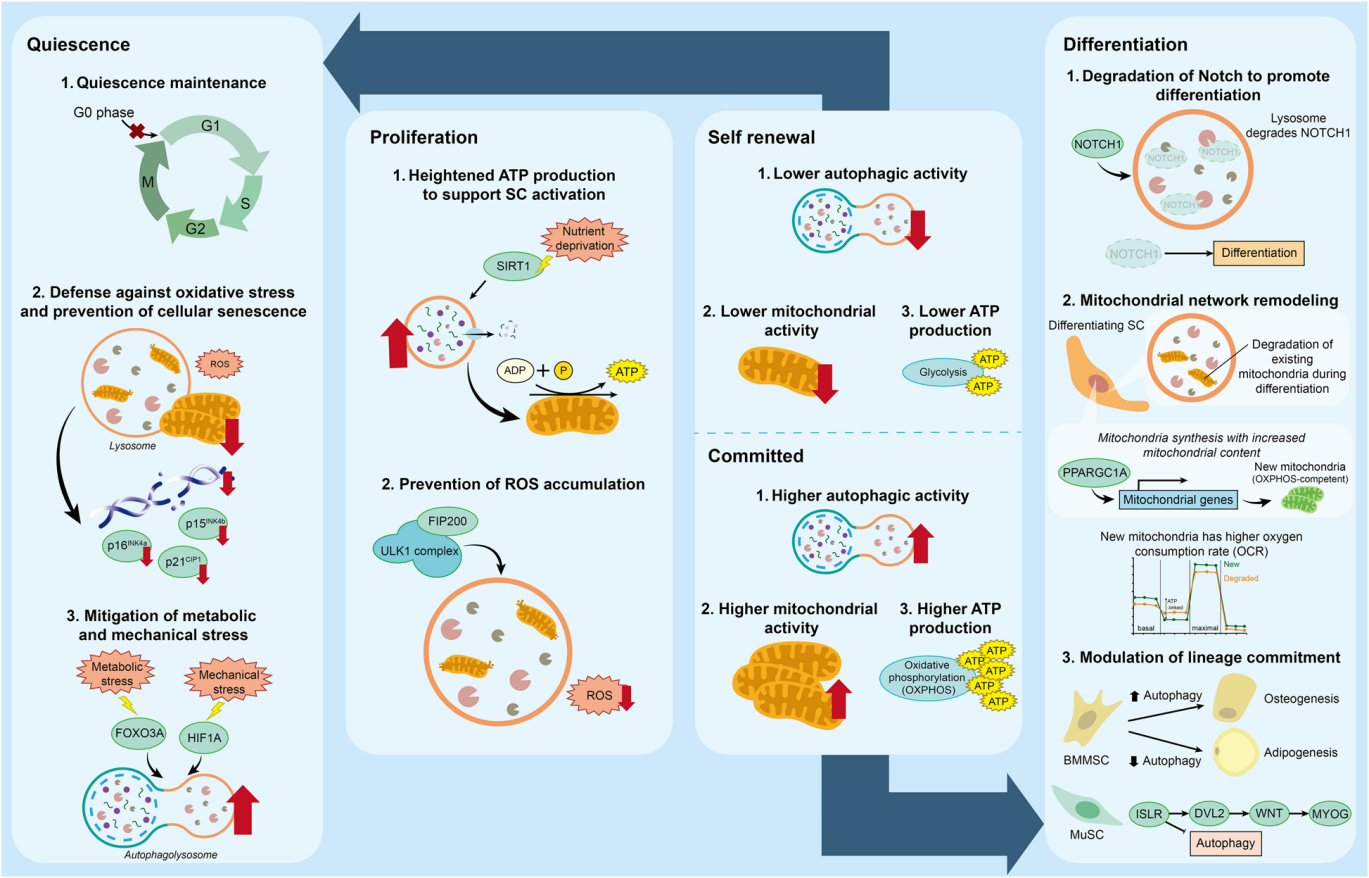
The role of autophagy in stem cell quiescence, proliferation, self-renewal, and differentiation. During SC quiescence, autophagy is crucial for maintaining the quiescent state, resisting stresses (including oxidative, metabolic, and mechanical stresses), and preventing cellular senescence. As SCs enter the proliferation phase, autophagic activity increases to supply adequate nutrients and ATP for SCs, while withstanding oxidative stress. Upon SC fate determination, self-renewing SCs exhibit lower autophagic activity compared to committed SCs. In differentiated SCs, elevated autophagy is essential for degrading Notch, remodeling the mitochondrial network, enhancing energy production through oxidative phosphorylation (OXPHOS), and guiding specific lineage commitment

Autophagy is vital in maintaining stemness and protecting quiescent SCs against metabolic and oxidative stresses (Fig. 2). Different types of quiescent SCs demonstrate varying levels of autophagy, which will be discussed in this section.

Quiescent NSCs (qNSCs) express high levels of mRNA encoding lysosomal factors, including cathepsins (CtsA, CtsB, and CtsF), and lysosomal membrane protein LAMP1 (Kobayashi et al. 2019). TFEB (Transcription Factor EB), a master regulator of lysosomal biogenesis, is also activated in qNSCs. From the histological perspective, qNSCs contain more lysosomes than activated NSCs (aNSCs). During the initial establishment of qNSCs, protein aggregates accumulate within the cellular compartments (Calatayud-Baselga et al. 2023). Consequently, qNSCs acquire more autophagosomes, increase expression of aggrephagy receptor TAX1BP1 (Tax-1 binding protein 1) and lysosomal marker LAMP2, and activate the AMPK (AMP-activated protein kinase)/ULK1 complex to enhance the autophagy machinery. Mesenchymal stem cells (MSCs) interact with the niche sensory nerves through fibroblast growth factor 1 (FGF1) (Pei et al. 2023). FGF1, secreted by sensory nerves, binds to the FGF receptor (FGFR) expressed by MSCs, activating p-JNK (c-Jun NH 2 -terminal kinase) and mTOR signaling pathways, and ultimately upregulating autophagy. This interaction is essential in maintaining MSC homeostasis and its disruption results in the loss of MSCs and the lineage osteogenic progenitor cells. Escargot (Esg)-positive quiescent intestinal stem cells (qISCs) demonstrate a high number of ATG8a signals and increased autophagic activity compared to the differentiated cells (Nagy et al. 2018). Depletion of autophagy results in an increased level of phosphorylated histone H2A variant γ-H2Av, a marker of DNA damage and cell cycle arrest.

In contrast, MuSCs, HSCs, and salivary gland stem cells (SGSCs) maintain low levels of autophagy during quiescence. Quiescent MuSCs maintain a basal autophagic flux to facilitate protein and organelle turnover (García-Prat et al. 2016b). Quiescent HSCs are characterized by a low mitochondrial membrane potential (MMP) and thus low energy production (Liang et al. 2020). Quiescent HSCs also contain larger lysosomes when compared to primed HSCs, indicating that hampered lysosomal activity leads to the accumulation of undigested materials. SGSCs have lower autophagic flux compared to progenitor cells, with a reduced SQSTM1 turnover observed (Orhon et al. 2022).

### ATG5 and ATG7 are critical to establish SC quiescence

ATG5 and ATG7 are two well-characterized proteins essential for autophagosome formation. Genetic ablation of *ATG5* or *ATG7* disrupts autophagy and compromises quiescence maintenance, significantly reducing the SC population.

Impaired autophagy in quiescent SCs triggers premature activation. For example, ATG5 knockout (KO) in quiescent HSCs induces abrupt proliferation (Borsa et al. 2024). These altered HSCs exhibit heightened metabolic activity, including increased amino acid uptake, sustained mTOR activation, and elevated protein synthesis. Similarly, BafA1-treated qNSCs, with inhibition of lysosomal functions, display features of aNSCs (Kobayashi et al. 2019). Morphologically, qNSCs transition from a flat shape to a bipolar shape. These altered qNSCs also exhibit a proliferating phenotype, as evidenced by increased Ki67 and EdU markers. Additionally, they express elevated levels of phosphorylated EGFR (Epidermal growth factor receptor), the active form of Notch1 receptor NICD, and cyclin D1, indicating that lysosomal function-depleted qNSCs are undergoing activation.

Autophagy deficiency induces premature aging phenotypes in SCs. *ATG7*-null MuSCs demonstrate an upregulation of senescence-associated markers, including p16^INK4a^, p21^CIP1^, and p15^INK4b^ (García-Prat et al. 2016a) These cells also exhibit elevated levels of ROS and Parkin, a marker of mitophagy-mediated mitochondrial degradation. Ablation of ATG5 (Jung et al. 2019) or ATG7 (Mortensen et al. 2011) in HSCs leads to aberrant mitochondrial accumulation, resulting in an increased mitochondrial membrane potential and enhanced superoxide production. Eventually, a significant increase in DNA damage is observed. Similarly, *Beclin1 *KO in ISCs leads to elevated DNA damage, as indicated by the increased levels of phosphorylated histone H2A variants (Nagy et al. 2018). These altered ISCs are susceptible to genotoxic stress and cell cycle arrest, ultimately entering replicative senescence. These studies collectively indicate that the loss of autophagy in SCs results in a cascade of detrimental effects, including the loss of quiescence, premature activation, and potential onset of senescence. Consequently, the stem cell pool is diminished, impairing self-renewal capacity and compromising the regenerative potential of tissues.

### Autophagy and mitophagy protect quiescent SCs against stress

One of the major roles of autophagy is to safeguard quiescent SCs against diverse environmental stresses. Studies have demonstrated that autophagy is instrumental in mitigating oxidative, metabolic, and mechanical stress. This cytoprotective mechanism enables quiescent SCs to preserve their stemness and maintain the integrity of the SC pool, thereby supporting long-term tissue homeostasis (Eliazer 2019).

Autophagy and mitophagy are essential for maintaining SC quiescence by mitigating oxidative stress. The canonical mitophagy pathway, primarily mediated by Pink1 and Parkin, is highly active in quiescent MuSCs (Cairns 2024). Mitophagy receptors, including FUNDC1, BNIP3L, BCL2L13 (BCL2 Like 13), and Ambra1 (Autophagy and Beclin 1 regulator 1), are also enriched in these cells. The colocalization of mitochondria with autophagolysosomes suggests ongoing mitochondrial degradation during quiescence. Long-term HSCs (LT-HSC), representing the most quiescent population of HSCs, exhibit minimal mitochondrial activity and mass (Vannini et al. 2016; Ho et al. 2017). In MSCs, irradiation leads to ROS accumulation and DNA damage (Hou et al. 2013). Enhancing autophagy by rapamycin supplementation reduces ROS levels and DNA damage, as assessed by dichlorofluorescin diacetate (DCF-DA) and γ-H2AX, respectively. These results collectively indicate that mitophagy is responsible for removing healthy and active mitochondria from quiescent SCs to sustain a low metabolic state and reduce oxidative phosphorylation. The clearance of mitochondria contributes to the suppression of ROS production, helping to protect quiescent SCs from oxidative stress.

Autophagy is upregulated as a cytoprotective mechanism when adult SCs experience metabolic stress. In the bone marrow environment, quiescent HSCs occasionally encounter fluctuations in growth factors and nutrient deprivation (Warr et al. 2013). Stressed HSCs promote the apoptotic program and are less potent to reconstitute their lineages. FOXO3A is found to be highly expressed in quiescent HSCs, where it primes HSCs for rapid induction of autophagy in response to stressors, protecting HSCs from apoptosis and cell death.

Mechanical stress protection in adult SCs is also mediated by autophagy through hypoxia-induced mechanisms. Nucleus pulposus-derived stem cells (NPSCs), localized in the intervertebral discs (IVD), often experience physical stresses such as compression and overload, compromising their differentiation potential and viability (He et al. 2021b). The hypoxic environment in the nucleus pulposus induces the expression of HIF1A (Hypoxia-inducible factor 1-alpha). HIF1A exerts cytoprotective effects by downregulating apoptosis-associated proteins and activity, improving NPSC survival. HIF1A specifically upregulates BNIP3 expression, which interacts with ATG7 to promote autophagy. Hmox1 (Heme oxygenase 1), a downstream target of HIF1A, catalyzes the degradation of pro-oxidant heme and also mediates autophagy.

To summarize, autophagy and mitophagy serve as critical cytoprotective mechanisms, allowing adult SCs to resist oxidative, metabolic, and mechanical stresses. These processes are essential for maintaining the quiescent stem cell population and preserving their regenerative functions.

## Autophagy regulation in stem cell proliferation

### Autophagy provides energy sources to support adult SC activation and proliferation, while preventing ROS accumulation

Autophagy plays a bioenergetic role during SC activation by supplementing ATP through cellular recycling (Fig. 2). Due to heightened metabolic demands during adult SC activation, the cellular energy status and nutrient availability change. Subsequently, Sirtuin1 (Sirt1), a nutrient sensor, is activated to deacetylate ATG7 and promote the induction of autophagy (Lee et al. 2008; Tang and Rando 2014). Autophagic activity is increased during MuSC activation, coinciding with an increase in ATP levels (Rodgerset al. 2014). The reduction in ATP content and the delay in MuSC activation due to autophagy inhibition can be partially rescued by supplementing exogenous pyruvate, highlighting the bioenergetic importance of autophagy in MuSC activation.

While elevated ATP synthesis is observed during SC proliferation, autophagy serves as a defense mechanism to prevent the accumulation of ROS generated by ATP production. RB1 inducible coiled-coil 1 (Fip200) is a scaffold protein that interacts with ULK1 complex to regulate autophagosome formation. *Fip200 *deficiency in NSCs causes p62 aggregation, which disrupts the function of superoxide dismutase (SOD), an antioxidant enzyme, and subsequently escalates superoxide and accumulates ROS (Wang et al. 2016a). Deletion of *ATG5 *(Asano et al. 2017) and *ATG7 *(Trentesaux et al. 2020) in ISCs also leads to the accumulation of ROS and increased apoptosis. The proliferative capacity of ISCs is significantly impaired. Conversely, antioxidant therapy could partially restore the impaired proliferation of ISCs and intestinal regeneration in models of depleted autophagy (Asano et al. 2017).

### Dysregulated autophagy increases apoptosis and decreases adult SC proliferation

The accumulation of ROS due to the absence of autophagy is a primary cause of oxidative stress and subsequent apoptosis. In adult neurogenesis, autophagy regulatory factors Ambra1 and Beclin1 (Becn1) are essential for the survival and proliferation of NSCs. Downregulation of these proteins in NSCs leads to reduced proliferation, increased apoptosis, and enhanced sensitivity to DNA damage-induced cell death (Yazdankhah et al. 2014). Similarly, autophagy-deficient ISCs exhibit increased DNA damage, cell cycle arrest (Nagy et al. 2018), and altered interactions with the microbiota through the p53-mediated pathway (Trentesaux et al. 2020). These dysregulated ISCs are ultimately cleared by JNK-mediated apoptosis (Nagy et al. 2018). Loss of *ATG5 *leads to a decline in the self-renewal capacity of SGSCs and an increase in apoptosis (Orhon et al. 2022). It is also observed in bone marrow-derived mesenchymal stem cells (BMMSCs) that impaired autophagy results in elevated ROS levels and activation of p53, which eventually promotes cell cycle arrest and apoptosis (Ma et al. 2018). Besides, enhancing autophagy through fasting could protect SCs from DNA damage and cell death (Trentesaux et al. 2020). These results collectively suggest that autophagy is crucial in preserving proper SC functions by maintaining genomic integrity and resisting cytotoxic stress.

## Autophagy regulations in stem cell fate decisions

SC fate determination directs proliferating SCs towards either self-renewal to replenish the SC pool or differentiation into specialized cell types (Fig. 2). Self-renewing cells maintain their stemness properties and primarily rely on anaerobic glycolysis for energy production. In contrast, differentiating cells have higher energy demands and thus shift to oxidative phosphorylation. Therefore, metabolic states and energy source transitions are considered potential markers of fate decisions.

Autophagy is proposed to influence SC fate decisions by modulating organelle degradation. During muscle regeneration, the self-renewing MuSCs exhibit low levels of autophagy, with only 9.6% cells being LC3-positive (Cairns et al. 2024). Conversely, the majority of differentiating MuSCs (71.8%) are LC3-positive. Inhibition of mitophagy leads to elevated ROS levels, triggering MuSCs to commit to differentiation (Fiacco et al. 2016). Introducing mitoTEMPO, a mitochondria-targeted superoxide scavenger, reduces the number of committed cells and restores the self-renewing MuSC population. Similar responses have also been observed in HSCs. The use of TMRM (tetramethylrhodamine methylester), a cellular dye that indicates mitochondrial polarization and oxidative phosphorylation, reveals that TMRM-high HSCs are more committed, while TMRM-low HSCs retain stemness (Vannini et al. 2016). In a differentiating environment, lowering HSC mitochondrial activity by chemically uncoupling the electron transport chain drives HSCs towards self-renewal. These findings indicate that autophagy modulates mitochondrial activity and thereby orienting SCs towards either self-renewal or commitment pathways.

Autophagy is also involved in determining the lineage commitment of SCs. In BMMSCs, the inhibition of autophagic activity leads to a decrease in osteogenic differentiation while promoting adipogenic differentiation (Ma et al. 2018). These results suggest that the precise regulation of autophagy is essential for achieving balanced differentiation and maintaining cellular homeostasis.

## Autophagy regulations in stem cell differentiation

### Autophagy regulates Notch signaling to facilitate adult SC differentiation

The Notch signaling pathway plays a central role in cell–cell interactions, thereby regulating SC differentiation. During SC differentiation, autophagy targets and degrades the Notch receptor by capturing membrane-associated Notch1 into ATG16L1-positive autophagosomal precursor vesicles (Fig. 2). The deficiency in *ATG16L1 *leads to aberrant Notch1 expression, which impairs NSC differentiation and consequently results in developmental retardation and altered neurogenesis (Wu et al. 2016). The Notch signaling pathway also participates in BMMSC differentiation. Induction of autophagy using rapamycin inhibits the proliferation of BMMSCs and promotes neuronal differentiation, as evidenced by the increased expression of neuron-specific markers, such as Neuron-specific enolase (NSE) and Microtubule-associated protein 2 (MAP2). Conversely, inhibition of autophagy by 3-methyladenine (3-MA) or CQ reduces apoptosis and neuronal differentiation but has no impact on proliferation (Li et al. 2016). These findings reveal a new intersection between autophagy and developmental pathways in regulating adult SC differentiation.

### Autophagy remodels the mitochondrial network to switch energy sources

Several types of SCs experience an energy shift from glycolysis to oxidative phosphorylation to meet the increased energy demands. The transition requires the remodeling of the mitochondrial network, which includes the clearance of existing mitochondria through mitophagy and reestablishing a dense filamentous mitochondrial network that encompasses higher oxygen consumption rates (OCRs) with higher content of OXPHOS components (Fig. 2). For instance, during skeletal myogenesis, Dynamin 1 like (Dnm1l)-mediated mitochondrial fission and SQSTM1-mediated mitophagy are upregulated. Besides, autophagic markers such as *Map1lc3a* (*Microtubule-associated proteins 1A/1B light chain 3A*) are also upregulated, followed by a significant increase in mitochondrial content through Peroxisome proliferator-activated receptor gamma coactivator 1-alpha (PPARGC1A)-mediated biogenesis. The use of autophagy inhibitors BafA1 or siRNA targeting *ATG5* and *SQSTM1 *hinders myogenic differentiation (Sin et al. 2016). Consistently, silencing VPS39 (Vacuolar protein sorting 39), an autophagy protein mediating the fusion of autophagosomes with lysosomes, results in reduced glucose uptake, lower autophagic flux, and impaired differentiation (Davegårdh et al. 2021). These findings uncover a critical role of autophagy in mitochondrial network remodeling and energy metabolism shift during stem cell differentiation.

### Fine-regulation of autophagy is critical for adult SC differentiation progression

Autophagy is fine-regulated by integrated signals, in which varying levels of autophagy can lead to distinct impacts on cell fate (Fig. 2). Excessive and deficient levels of autophagy are both detrimental to the metabolism and differentiation of SCs. Mild autophagy, which can be induced by moderate serum starvation, promotes cellular metabolism and differentiation in MuSCs. In contrast, severe autophagy leads to increased apoptosis, metabolic defects, and inhibition of MuSC differentiation. The AMPK/mTOR pathway was identified as a key regulator in this process, with mild autophagy enhancing the expression of the muscle differentiation markers Myoblast determination protein 1 (MyoD1) and Myosin heavy chain (MHC), as well as the adipogenic markers peroxisome proliferator-activated receptor gamma (PPARγ) and Lipoprotein lipase (LPL). In contrast, severe autophagy suppressed the expression of these markers (Wang et al. 2023).

Enhancing autophagy signaling via electrical stimulation (ES) promotes the differentiation of NSCs into mature neurons. By applying ES, the expression of mature neuronal markers such as β III tubulin (Tuj1) and MAP2, neurite outgrowth, brain-derived neurotrophic factor (BDNF) concentration, and electrophysiological activity were increased. RNA sequencing analysis has confirmed that ES promotes the differentiation of NSC-derived neurons by upregulating autophagy-associated genes, including those involved in autophagosome formation and autophagy flux regulation, and by activating key autophagy-regulating signaling pathways such as PI3K/Akt and mTOR. Notably, excessive autophagy induced by overly strong ES inhibited the NSC viability. Meanwhile, the use of autophagy inhibitors rescued the negative effects caused by excessive ES (He et al. 2021). These data suggest that optimal autophagic activity is required to support proper SC functions.

### The role of autophagy in tissue-specific differentiation

Autophagy not only universally suppresses Notch signaling and supplies energy to support SC differentiation but also activates tissue-specific pathways to mediate lineage commitment and differentiation.

Autophagy levels are highly correlated with hematopoietic potential. Sphingolipid metabolism modulates autophagy and the unfolded protein response (UPR) to regulate self-renewal and differentiation of HSCs. The sphingolipidase DEGS1 activates autophagy and the UPR through the inhibition of N-(4-hydroxyphenyl) retinamide (4HPR), which is crucial for maintaining the identity of HSCs. This regulation affects lineage commitment, indicating a link between sphingolipid metabolism and HSC fate determination (Xie et al. 2019). In contrast, impaired autophagy by depleting ATG5 in endothelial cells disrupts the endothelial-to-hematopoietic transition (EHT). Autophagy also modulates the distribution and function of nucleolin, thereby the ribosome biogenesis processes (Liu et al. 2024). These are crucial processes in the development and maturation of hematopoietic stem cell precursors (HSC I). Furthermore, the conditional deletion of *ATG7 *in the mouse hematopoietic system, leads to the loss of HSC functions and aberrant proliferation of myeloid cells, resembling acute myeloid leukemia (AML) in humans. Loss of autophagy results in progenitor expansion, increased mitochondrial stress, and elevated ROS levels, all of which contribute to DNA damage and uncontrolled cell proliferation (Gomez-Puerto et al. 2016). These findings emphasize the significance of autophagy in HSC development.

MuSC differentiation and muscle regeneration is regulated by Wnt signaling with the interplay of autophagy. ISLR (Immunoglobulin superfamily protein immunoglobulin superfamily consisting of the leucine-rich repeat) protein modulates Dvl2 (Dishevelled-2) protein, a key component of the canonical Wnt signaling pathway. ISLR stabilizes Dvl2 by inhibiting the autophagic system, thereby promoting myogenic differentiation. The subsequent activation of canonical Wnt signaling regulates myogenic factors such as Myogenic factor 4 (MyoG). This indicated a complex interaction between autophagy and stem cell differentiation during muscle regeneration (Zhang et al. 2018).

Modulating autophagic activities influences the differential potential of MSCs. Undifferentiated MSCs accumulate a large number of autophagosomes, which are rapidly degraded during differentiation (Nuschke et al. 2014). The balance of autophagy is crucial for the effective differentiation of MSCs into the osteogenic and adipogenic lineages. For instance, the activation of AMPK initiates the autophagic process and promotes early osteogenic differentiation (Pantovic et al. 2013). Conversely, the supplementation of autophagy inhibitor 3-MA into young BMMSCs mimicked the aged phenotype, impaired their osteogenic potential, and enhanced their adipogenic capacity (Ma et al. 2018). These results suggest that autophagy can redirect the differentiation potential of MSCs and has potential implications for regenerative medicine (Morganti et al. 2019).

## Autophagy during aging

**Fig. 3:**
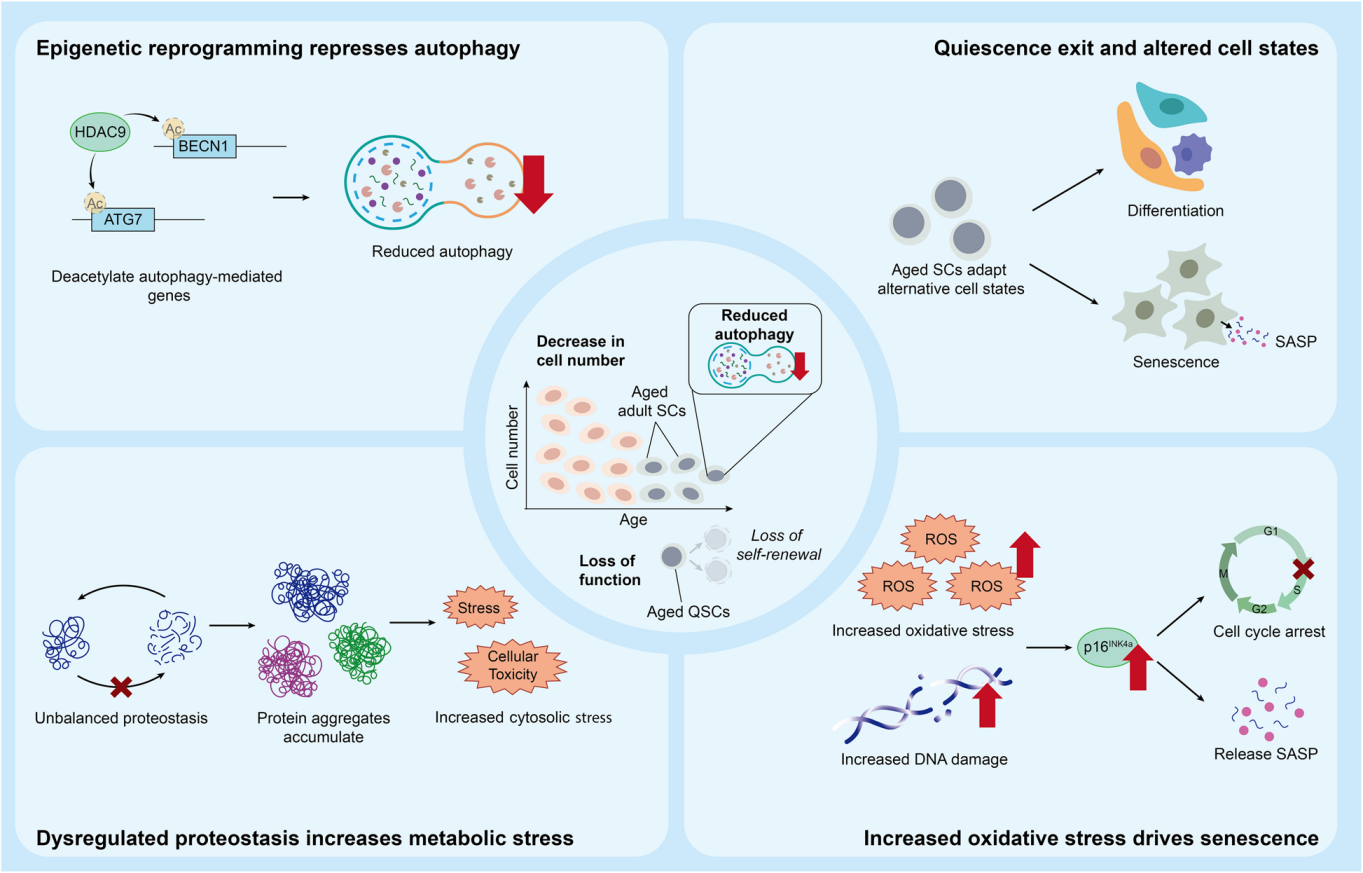
The relationship between reduced autophagy and stem cell dysfunction during aging. During aging, the epigenomic profile of aged SCs undergoes reprogramming that reduces autophagic activity. This reduction in autophagy leads to an imbalance in proteostasis and an increase in oxidative and cytosolic stresses. Eventually, aged SCs either differentiate or enter the state of senescence, exhibiting diminished regenerative capacity

Aging is a complex biological process marked by the gradual deterioration of cellular functions and tissue homeostasis. As individuals age, the SC niche is distorted with the functionality and quantity of SCs declining, thereby impairing tissue homeostasis and regeneration (Miyamoto et al. 2007). Importantly, studies demonstrated that diminished autophagic activity is associated with the functional decline of adult SCs (Fig.3).

Epigenetic reprogramming in aged adult SCs alters the autophagic flux. In aged MSCs, the expression of histone deacetylase HDAC9 is upregulated (Zhang et al. 2020). HDAC9 binds and deacetylates autophagy-related genes, such as *ATG7* and *Becn1*, eventually suppressing and impairing the autophagic processes. HDAC9 inhibition is able to restore the balance of cellular differentiation and enhance autophagy by acetylating the promoter regions of the corresponding genes.

Proteostasis is dysregulated with diminished autophagy during aging. The Bag3-dependent transportation mechanism facilitates the aggregation of misfolded proteins and promotes aggrephagy (Zhang et al. 2022a). During aging, the expression of Bag3 is reduced, resulting in impaired aggresome formation and aggrephagy (Myers et al. 2018). The decline in autophagic flux is also coupled with increased proteasome activity, leading to imbalanced proteostasis. Harmful protein aggregates are retained in HSCs which adversely affects their functions (Chua et al. 2023).

Reduced autophagic activity also hinders SCs from obtaining energy and nutrient supplementation. The lowered autophagic activity is correlated with the upregulation of amino acid transporter proteins, namely the Solute carrier (SLC) family (Borsa et al. 2024). The SLC family aims to compensate for the adverse effects of diminished autophagic activity by enhancing amino acid absorption and safeguarding necessary nutrients to support HSCs. While the compensatory mechanisms prolonged HSC survival, the constitutive expression of the SLC family results in sustained activation of the mTOR pathway, which is detrimental to the maintenance of the adult SC population. Eventually, the HSC population diminishes with lowered regenerative potential.

The reduction in autophagy during aging increases oxidative stress and drives adult SCs into senescence. Sirtuin 3 (Sirt3) is a key mitochondrial protein activated during autophagy and is responsible for ameliorating oxidative stress and delaying HSC aging (Fang et al. 2020). During aging, the decline in autophagy is associated with decreased expression of Sirt3. Increased oxidative phosphorylation, quiescence exit and myeloid differentiation are observed in HSCs (Ho et al. 2017). Additionally, impaired mitophagy in aged MuSCs results in the accumulation of damaged proteins and cellular organelles, including mitochondria (García-Prat et al. 2016b). A high level of ROS persists without proper recycling and reconstituting healthy mitochondria. The elevated ROS level disrupts PRC1-mediated H2A monoubiquitylation at the INK4a locus, leading to the induction of p16^INK4A^ expression and driving MuSCs into senescence. Similarly, cellular senescence is observed in the replicative aged ex vivo periodontal ligament stem cells (PDLSCs), with decreased proliferation, increased expression of senescence-associated proteins, and enhanced senescence-associated-β-galactosidase (SA-β-gal) activity (Tan et al. 2021).

Enhancing autophagic activity can ameliorate aged-related functional decline of adult SCs. It is observed that a subset of aged HSCs possesses an intrinsically high autophagic capacity, in which these HSCs maintain low metabolic activity and robust regenerative potential (Ho et al. 2017). Therefore, extrinsic approaches in increasing autophagy are adopted. For instance, BCL2 binds the inactive form of Becn1 to inhibit autophagy. The*BECN*^*F121A/F121A*^mutation reduced the affinity towards BCL2, thereby enhancing autophagy. The mutated mouse model exhibited sustained autophagy and preserved NSC functions, with improved self-renewal and differentiation capacity. On the contrary, melatonin (MLT) interacts with specific melatonin receptors, which inhibits the PI3K/Akt/mTOR signaling pathway (Tan et al. 2021). The introduction of MLT restores impaired autophagy in aged SCs and ameliorates the senescent condition. In Drosophila ISCs, deficiency in *Beclin 1* orthologue (*ATG6*) induced age-related hyperproliferation, centrosome amplification, and DNA damage. Introduction of metformin activates the *ATG6*-dependent pathway and suppresses the AKT/mTOR pathway, which lowers the age-associated accumulation of DNA damage and oxidative stress markers in old ISCs (Na et al. 2018). AMPK activation or phosphomimetic p27^Kip1^ expression rescued aged MuSC dysfunction by restoring autophagy, reducing apoptosis/senescence, and enhancing transplantation efficacy (White et al. 2018). These findings underscore the potential of targeting autophagy to ameliorate the age-related deterioration of adult SC functions.

## Therapeutical insights

Autophagy is closely correlated to adult SC activity. It does not only finely regulate the physiological processes of SCs but also serves as a quality control system to prevent the accumulation of dysfunctional components. Recent studies demonstrated that enhancing autophagic activity during disease and aged conditions hold therapeutic implications.

Dietary interventions such as TRF and CR (calorie restriction) aim to extend lifespan and healthspan. These dietary interventions result in periodic nutrient deprivation, which triggers the upregulation of autophagy. Studies indicated that fasting regimens potentially promote SC activation and regeneration by SPD (Spermidine)-eIF5A (Eukaryotic translation initiation factor 5A)-mediated autophagy (Chrisam et al. 2015; Hofer et al. 2024; Zhang et al. 2024). SPD, a naturally occurring polyamine, hypusinates eIF5A and mediates the myogenic transcription factor MyoD1 to promote MuSC activation (Zhang et al. 2024). During fasting, SPD and hypusinated eIF5A are upregulated, ultimately enhancing ATG3 translation and promoting autophagy (Lubas et al. 2018). In the context of disease, administering SPD in collagen VI-null myopathic models helped to clear damaged proteins and organelles, thereby alleviating myopathic defects (Chrisam et al. 2015). The results suggest that fasting could potentially ameliorate disease phenotypes. On the other hand, aged epidermal SCs and MuSCs re-establish their circadian transcriptome when they experience tissue-specific stresses, including DNA damage and reduced autophagy. The practice of CR not only enhances autophagy but also mitigates the altered circadian gene expression and SC function, suggesting the role of CR in resynchronizing cellular metabolism with circadian rhythm. The finding emphasizes the potential of dietary interventions to combat age-related decline in autophagy and SC rhymicity (Solanas et al. 2017). While the direct relationship between fasting regimens and SC activation or tissue regeneration has not been fully elucidated, these studies collectively proposed that fasting-induced autophagy could indeed serve as a therapeutic strategy for aging-related disorders.

Reducing oxidative stress is another promising approach in enhancing autophagy and restoring mitochondrial functions. NAD^+ ^(Nicotinamide adenine dinucleotide) is a coenzyme supporting to the antioxidant defense mechanism (Jo et al. 2020). During aging, the levels of NAD^+^ decline, leading to impaired mitochondrial functions and senescence in MuSCs. Treating with NR (Nicotinamide riboside), an NAD^+^ precursor, activates the mitochondrial unfolded protein response (UPR^mt^) and enhances autophagy, thereby restoring the vitality of aged MuSCs and improving muscle regeneration. Besides, the suppression of AKT/mTOR pathway by metformin also results in lower oxidative stress-correlated DNA damage, as demonstrated by reduced H2AX and 8-oxo-dG levels in Drosophila ISCs (Na et al. 2018). These results highlighted the importance of withstanding oxidative stress with autophagy in promoting SC functions, and suggests that NAD^+ ^and metformin supplementation could be potential treatments for ameliorating aging phenotypes and enhancing tissue repair (Zhang et al. 2016).

Recent discoveries shed light on the application of anti-autophagy drugs in treating cancers. Cancer stem cells (CSCs) exhibit characteristics similar to adult SCs, such as self-renewal and differentiation potential. They are present in various types of cancer, including breast, brain, and lung, and serve as the critical drivers of tumor initiation, progression, and recurrence (Leon et al. 2016; Marcucci et al. 2017; Prasetyanti and Medema 2017; Saw et al. 2024). CSCs typically exhibit elevated levels of basal autophagy compared to non-cancer SCs (Levine and Kroemer 2019). When the core ATG genes are knocked down in breast CSCs, it significantly hinders their ability to self-renew in vitro. Moreover, upon xenografting these genetically manipulated CSCs into mice, tumor growth is notably impaired (Boya et al. 2018). It is observed that increased autophagy promotes the invasion and migration of glioblastoma multiforme SCs by regulating mitochondrial function and metabolic features (ATP and lactate) (Galavotti et al. 2013). Autophagy also contributes to the resistance of CSCs to metabolic stress or genetic instability induced by radiation-induced damages. Importantly, increased autophagy is associated with the gradual acquisition of drug resistance in CSCs during cancer therapy. Autophagy enhances the survival of CSCs by inhibiting apoptosis, adapting to metabolic stress, preserving pluripotency, evading immunosurveillance, and facilitating metastasis, which ultimately reduces the effectiveness of cancer therapies (Kim et al. 2017; Buccarelli et al. 2018; You et al. 2019). Therefore, targeting and depleting autophagy in CSCs could be a promising strategy to delay cancer progression and improve the effectiveness of therapeutics. Studies discovered that the use of clinically approved autophagy inhibitors (CQ and HCQ) effectively increases the sensitivity of CSCs to the cytotoxic effects of chemotherapeutic drugs and radiotherapy (Chude and Amaravadi 2017; Nazio et al. 2019; Rothe et al. 2019; Smith and Macleod 2019). The inhibition of the protective autophagic processes that CSCs employ to survive therapeutic stresses allowing them to be more vulnerable to treatment-induced cell death. However, the use of these inhibitors remains challenging. For instance, CQ has been associated with significant toxicity in some cases. Not all autophagy can be inhibited by CQ in vivo, suggesting a need for more effective autophagy inhibitors for clinical applications (Skendros et al. 2017). Nevertheless, the development of novel cancer-targeted autophagy inhibitors, when combined with existing cancer therapies, offers a promising strategy for enhancing the effectiveness of cancer treatments.

## Conclusions and perspectives

Adult SCs play crucial roles in maintaining homeostasis and tissue regeneration. The maintenance of quiescence, activation, self-renewal, and differentiation of adult SCs are precisely regulated by the microenvironment and intracellular signals. The dysregulation of adult SCs during aging contributes to the development of aging-associated diseases. A more comprehensive understanding of the regulatory mechanisms governing SC regulation will provide novel insights into the development of clinical treatments for ameliorating aging phenotypes and extending lifespan and health span. Here, we summarize the recent studies elucidating the role of autophagy in modulating SC functions and states. Autophagy, as a cellular degradation mechanism that clears damaged and misfolded substrates and recycles cellular components, strongly correlates to every cell state of SCs.

The maintenance of adult SC quiescence is highly dependent on autophagy to recycle fatty acids and proteins, thereby alleviating macromolecular damage and preventing quiescence exit (Nagy et al. 2018; Yazdankhah et al. 2014). Although numerous studies demonstrated varying levels of basal autophagic flux across different quiescent adult SCs, it is indisputable that the absence of autophagy leads to diminished stemness and exhaustion of the SC pool (García-Prat et al. 2016b; Nagy et al. 2018; Kobayashi et al. 2019; Orhon et al. 2022). Notably, quiescent SCs with depleted autophagy exhibit characteristics of SC activation. This observation suggested that quiescent SCs are more prone to activation to compensate for the loss of nutrients and clearance of accumulated damaged organelles.

Activated SCs re-enter the cell cycle and proliferate, and this process is accompanied by increased autophagic activity. During activation, the chromatin structure is more open, and the SCs undergo substantial metabolic reprogramming (Dong et al. 2022). Autophagy is responsible for degrading cellular components to provide nutrients and more ATP, supplementing activated SCs with increasing energy demand (Tang and Rando 2014). However, other studies have suggested an opposite effect in which the overall level of autophagy was decreased upon activation (Nagy et al. 2018; Leeman et al. 2018). This suggests that alternative energy sources might also promote SC activation. Furthermore, extensive ATP synthesis results in increased ROS generation. ROS accumulation leads to oxidative damage and mitochondrial dysfunction. Autophagy is crucial to eliminate ROS and sustain mitochondrial homeostasis (Levine and Kroemer 2019). Strikingly, ROS is also critical for SC activation (Vitale et al. 2017). The maintenance of the ROS balance is therefore important for proper SC functions. While the process by which autophagy supplies energy for SC activation is not universal, more studies are looking to decipher the role of autophagy-mediated metabolites in regulating SCs.

Autophagy contributes to organelle remodeling in SC differentiation by reconstituting the complex mitochondrial network to facilitate metabolic reprogramming (Gong et al. 2015). Differentiated SCs often exhibit enhanced metabolic demands. While an increase in autophagy has been observed in differentiating MuSCs (Cairns et al. 2024), this outcome appears contradictory to studies that demonstrated the suppression of mitophagy paradoxically promoted SC differentiation (Fiacco et al. 2016). These studies suggested that altered metabolic states and elevated ROS levels appear to be a potential regulator of SC differentiation, which aligns with its role in inhibiting SC proliferation (Fiacco et al. 2016; Vannini et al. 2016). Besdes, the fine regulation of autophagy is crucial to direct lineage commitment and modulate SC function (Ma et al. 2018). Autophagy regulates SC differentiation by modulating the Notch signaling pathway (Li et al. 2016; Wu et al. 2016). The enhancement and attenuation of autophagy appear to guide differentiation towards specific lineages in multipotent SCs. It is also observed that moderate levels of autophagy are promoting MuSC differentiation, but excessive autophagy is detrimental (Wang et al. 2023). Given the intricate regulation of autophagy during SC activation and differentiation, and the heterogeneity of the SC populations, single-cell omics and spatiotemporal techniques offer powerful tools to decipher how SCs interact with the niche to modulate autophagic activity and determine cell fates.

Aging SCs are dysregulated in which the quantity and functionality significantly decline. These aging SCs exhibit impaired mitochondrial functions, accumulated oxidative stress, and express cellular senescence markers (Zeng et al. 2023). Concurrently, a decline in autophagic flux accelerates this process. Enhancing autophagy in aged SCs facilitated the clearance of dysfunctional mitochondria to promote efficient oxidative phosphorylation and lower oxidative damage by reducing ROS levels (Dikic and Elazar 2018). This process preserves the SC pool in older individuals, with such effects appearing to be achievable through the enhancement of mitochondrial functions (Zhang et al. 2016). Although the decline in SC functions due to aging is well-established, considering the broad regulation of cellular homeostasis by autophagy, targeting the modulation of autophagy in specific tissue or cell types is essential for translatable clinical applications. Increasing studies demonstrated the potential effects of dietary interventions on SC functions. CR activates autophagy through AMPK and are associated with subsequent metabolic shifts in SCs, promoting regeneration (Chrisam et al. 2015; Hofer et al. 2024; Zhang et al. 2024). IF and ketogenic diets constitute effective dietary patterns for fine-tuning autophagy. Nevertheless, future research should prioritize the understanding of the dual roles of autophagy in promoting and suppressing SC functions to develop targeted strategies in suppressing CSC growth and combating aging.

To summarize, the regulations of autophagy in the cellular processes and cell states of adult SCs are strongly correlated, yet highly complex. Deciphering the detailed molecular mechanisms provides clinical insights into the understanding of aged SC- and cancer-related pathologies, thereby proposing lifestyle interventions and treatments in ameliorating disease phenotypes and eventually promoting longevity.

As the understanding of autophagy's role in adult SC homeostasis, aging, and disease therapy continues to evolve, several critical questions and technical needs emerge that warrant further exploration.

Employing single-cell omics technologies to dissect autophagy heterogeneity within SC subpopulations can reveal critical insights into how different SCs respond to autophagy modulation (Giustacchini 2017; Shan 2022). This approach can help identify specific autophagy-related genes or pathways that are differentially regulated in various SC types, providing a more nuanced understanding of their roles in homeostasis and disease. Moreover, the development of advanced live-imaging techniques to visualize autophagy dynamics within SC niches is essential (Morikawa and Takubo 2017). Such technologies can provide insights into the spatiotemporal regulation of autophagy and its impact on SC fate decisions in real-time. This could enhance our understanding of how autophagy influences SC behavior in their native environments, potentially leading to breakthroughs in tissue engineering and regenerative therapies.

One of the foremost challenges is developing strategies to selectively target autophagy in tissue-specific SCs without inducing systemic side effects (Moors et al. 2017). This is crucial for therapeutic applications, especially in regenerative medicine and cancer therapy, where autophagy modulation can have profound effects on SC behavior and tissue regeneration. Future studies should focus on identifying specific markers or pathways that can be exploited for targeted interventions. Despite the promising potential of autophagy modulation in therapeutic contexts, several clinical challenges remain. These include understanding the dual roles of autophagy in tumor suppression and promotion, as well as the need for precise dosing and timing of autophagy-targeting agents to avoid adverse effects (Singh et al. 2018; Yun et al. 2010). Addressing these challenges will be crucial for translating autophagy research into effective clinical therapies for aging and cancer (Nazio et al. 2019).

In conclusion, addressing these outstanding questions and technical needs will be essential for advancing the field of autophagy research in adult stem cells. By leveraging cutting-edge technologies and interdisciplinary approaches, we can unlock the therapeutic potential of autophagy in regenerative medicine and age-related diseases.

## Data Availability

Not applicable.

## Abbreviations

3-MA: 3-Methyladenine
4HPR: N-(4-hydroxyphenyl) retinamide
aNSCs: Activated neural stem cells
AML: Acute myeloid leukemia
AMPK: AMP-activated protein kinase
ATG: Autophagy-related protein
BafA1: Bafilomycin A1
BDNF: Brain-derived neurotrophic factor
BMMSCs: Bone marrow-derived mesenchymal stem cells
BNIP3: BCL2/adenovirus E1B interacting protein 3
Becn1: Beclin1
CHIP: C-terminus of Hsc70 interacting protein
CHMP4B: Charged Multivesicular Body Protein 4B
CMA: Chaperone-mediated autophagy
CQ: Chloroquine
CR: Caloric restriction
DCF-DA: Dichlorofluorescin diacetate
Dvl2: Dishevelled-2
EHT: Endothelial-to-hematopoietic transition
eIF5A: Eukaryotic translation initiation factor 5a
ER: Endoplasmic reticulum
ES: Electrical stimulation
ESCRT: Endosomal sorting complexes required for transport
Esg: Escargot
FGF1: Fibroblast growth factor 1
FGFR: Fibroblast growth factor receptor
Fip200: RB1 inducible coiled-coil 1
FOXO: Forkhead box O
FUNDC1: FUN14 domain containing 1
HCQ: Hydroxychloroquine
HDAC9: Histone deacetylase 9
HIF1A: Hypoxia-inducible factor 1-alpha
Hmox1: Heme oxygenase 1
HSCs: Hematopoietic stem cells
HSCA8/HSC70: Heat shock cognate protein 70
ISCs: Intestinal stem cells
ISLR: Immunoglobulin superfamily containing leucine-rich repeat protein
IVD: Intervertebral discs
KO: Knockout
LAMP2A: Lysosomal associated membrane protein 2A
LC3: Microtubule-associated protein 1 light chain 3
LIF: LC3-interacting region
LPL: Lipoprotein lipase
MAP2: Microtubule-associated protein 2
MHC: Myosin heavy chain
MLT: Melatonin
MMP: Mitochondrial membrane potential
MSCs: Mesenchymal stem cells
mTORC1: Mammalian target of rapamycin complex 1
MuSCs: Muscle stem cells
MVBs: Multivesicular bodies
MyoD1: Myoblast determination protein 1
MyoG: Myogenic factor 4
NAD^+^: Nicotinamide adenine dinucleotide
Nix/BNIP3L: BCL2/adenovirus E1B interacting protein 3-like
NPSCs: Nucleus pulposus-derived stem cells
NR: Nicotinamide riboside
NSCs: Neural stem cells
NSE: Neuron-specific enolase
OXPHOS: Oxidative phosphorylation
P62/SQSTM1: Sequestosome 1
Pink1: PTEN-induced putative kinase 1
Prkn: Parkin RBR E3 ubiquitin protein ligase
PDLSCs: Periodontal ligament stem cells
PE: Phosphatidylethanolamine
PI3K: Phosphoinositol-3 kinase
PI3P: Phosphatidylinositol 3-phosphate
PPARGC1A: Peroxisome proliferator-activated receptor gamma coactivator 1-alpha
PPARγ: Peroxisome proliferator-activated receptor gamma
PRC1: Protein regulator of cytokinesis 1
qISCs: Quiescent intestinal stem cells
qNSCs: Quiescent neural stem cells
RAB: Ras-associated binding protein
ROS: Reactive oxygen species
SA-β-gal: Senescence-associated-β-galactosidase
SASPs: Senescence-associated secretory proteins
SCs: Stem cells
SGSCs: Salivary gland stem cells
Sirt1: Sirtuin 1
Sirt3: Sirtuin 3
SLC: Solute carrier
SNARE: Soluble N-ethylmaleimide-sensitive factor attachment protein receptors
SOD: Superoxide dismutase
SPD: Spermidine
TEM: Transmission electron microscopy
TFEB: Transcription factor EB
TMRM: Tetramethylrhodamine methylester
TRF: Time-restricted feeding
Tuj1: β III tubulin
ULK1: Unc-51 like autophagy activating kinase 1
UPR: Unfolded protein response
UPR^mt^: Mitochondrial unfolded protein response
VPS4: Vacuolar Protein Sorting 4 Homolog A
VPS34: Vacuolar protein sorting 34
VPS39: Vacuolar protein sorting 39
WIPI2: WD repeat domain, phosphoinositide interacting 2
